# Improving Performance of the PRYSTINE Traffic Sign Classification by Using a Perturbation-Based Explainability Approach

**DOI:** 10.3390/jimaging8020030

**Published:** 2022-01-30

**Authors:** Kaspars Sudars, Ivars Namatēvs, Kaspars Ozols

**Affiliations:** Institute of Electronics and Computer Science, Dzerbenes Str.14, LV-1006 Riga, Latvia; kaspars.sudars@edi.lv (K.S.); kaspars.ozols@edi.lv (K.O.)

**Keywords:** explainable AI, convolutional neural network, network compression

## Abstract

Model understanding is critical in many domains, particularly those involved in high-stakes decisions, e.g., medicine, criminal justice, and autonomous driving. Explainable AI (XAI) methods are essential for working with black-box models such as convolutional neural networks. This paper evaluates the traffic sign classifier of the Deep Neural Network (DNN) from the Programmable Systems for Intelligence in Automobiles (PRYSTINE) project for explainability. The results of explanations were further used for the CNN PRYSTINE classifier vague kernels’ compression. Then, the precision of the classifier was evaluated in different pruning scenarios. The proposed classifier performance methodology was realised by creating an original traffic sign and traffic light classification and explanation code. First, the status of the kernels of the network was evaluated for explainability. For this task, the post-hoc, local, meaningful perturbation-based forward explainable method was integrated into the model to evaluate each kernel status of the network. This method enabled distinguishing high- and low-impact kernels in the CNN. Second, the vague kernels of the classifier of the last layer before the fully connected layer were excluded by withdrawing them from the network. Third, the network’s precision was evaluated in different kernel compression levels. It is shown that by using the XAI approach for network kernel compression, the pruning of 5% of kernels leads to a 2% loss in traffic sign and traffic light classification precision. The proposed methodology is crucial where execution time and processing capacity prevail.

## 1. Introduction

Road traffic today is considered a complex and dynamic environment, where safety depends on several interrelated factors. When these factors are not taken into account, interactions occur that lead to road accidents [1]. The main reasons that lead to accidents are the characteristics of the drivers, the vehicle itself, and the road conditions [2]. One of the modern concepts related to road infrastructure is "self-explaining roads" [3]. The concept aims to provide the driver or the visual system of a self-driving car with information about the upcoming situation in a comprehensible, faithful, and trustworthy manner by using various measures related to traffic signs and road markings. Consideration of features is an integral part of the road control infrastructure and provides the driver with the necessary information, warns of road regulations, and ensures the safety of pedestrians. 

For autonomous driving, accurate and robust perception of traffic signs are essential for motion planning and discrimination capability. However, traffic sign detection and classification are still a challenge due to the following reasons: (1) traffic signs are easily confused with other objects in road scenes; (2) weather conditions, time of day, refection, and occlusions reduce classification performance; (3) size, shape, and colour of traffic signs; (4) slight inter-class variance due to similar appearance of signs [4].

As a result, traffic sign detection and classification are extensively studied in the computer vision community. Recently, the availability of large annotated datasets [5,6] and the improvement of computational performance with powerful GPU cards [7] have shown good results using convolution neural networks (CNNs) [8,9]. In order to classify all specific classes of traffic signs and lights, a classifier based on a CNN architecture from the Horizon 2020 ECSEL-JU project “Programmable Systems for Intelligence in Automobiles” (CNN PRYSTINE) was used for our experiment [10]. Despite the success of this approach, the existing CNN RRYSTINE classifier is still inadequate because it provides classification output that does not tell us what information in the input causes it to make its decisions. It is well known that CNNs consist of a highly complex internal structure and are therefore very difficult to explain due to their black-box nature. It is challenging to understand what exactly is going on in each layer. It is also known that during training, each layer gradually extracts higher-level features of the image until the final layer essentially predicts what the image shows. We have focused on using explainable AI (XAI) to identify novel higher-level patterns and detections and develop more precise classification strategies to overcome these limitations. In addition, the explanations ideally allow us to understand the model’s reasoning behaviour and why the model predicted explicit decisions, such as classifying the traffic sign in a specific manner or associating certain features with CNN performance.

Despite these advantages, CNN models require a significant amount of resources, such as processing capacity, energy, bandwidth, and storage capacity. Therefore, various CNN compression methods have been proposed in the existing literature to address these shortcomings, such as network pruning, sparse representation, bits precision, knowledge distillation, and miscellaneous [11]. The key reason to address this problem is to find a suitable method for CNN compression using the XAI approach. 

In this study, we investigate how model-level explainability can be used for network pruning and how pruning affects prediction precision. We show that the perturbation-based methods can be used to explain CNN decisions and can be used as a perspective tool to compress CNNs with a minimal trade-off in precision.

The following section discusses the perturbation-based explanation methods that are appropriate for evaluating network parameters. Section 3 gives an overview of the CNN PRYSTINE network architecture used for traffic sign and traffic light classification. Section 4 describes the mathematical background for the proposed methodology. Section 5 shows the results of the experiment. Finally, Section 6 presents the discussion and future directions as well as conclusions.

## 2. Perturbation-Based Methods

For black-box models like CNNs, the prediction process for a given model must be transparent, especially for decisions where the stakes are high, and explain why and how such outcomes occur. Current studies on algorithmic explanations for predictive models can be divided into three main approaches: attribution, distillation, and intrinsic. Attribution focuses on measuring attribution or feature relevance scores. The distillation is concerned with reducing the complexity of models by transforming them into simple, easily understandable surrogate models. Finally, the intrinsic approach integrates the inner states of the deep networks or modular algorithms to justify the model. First, the attribution approach can be divided into three subcategories: perturbation-based, functional, and structural explanations. The fundamental methods of the first subcategory are analysed here, and the most appropriate one was selected to support our experimental methodology.

The idea of perturbation-based explanations is to compute the attribution of features in a given model by simulating a lack of knowledge about the value of the feature or features [12]. In other words, perturbation methods attempt to evaluate attributions or feature relevance by testing the model’s response to feature removal, masking or altering and measuring the corresponding feature relevance values. In computer vision, attributions are visualized as heatmaps showing the influence on the features of the target output for each input feature. Perturbation-based explanations are often used with arbitrary prediction models and support individual predictions (local explanation). In image processing, these methods express an explanation by manipulating the input image and/or activations of a CNN [13]. If a perturbation highlights image regions, it has an easily explainable meaning, i.e., manipulating this region in the current input will significantly affect the prediction of the model. They have the advantage of a straightforward explanation, as they directly measure the marginal effect of some input features on output [14]. In this case, perturbation-based methods only require the propagation of one forward and/or backward pass through the CNN to generate a visualization of a heatmap. Understanding the visual perception aspects captured in a deep model has become particularly important for explaining deep networks. In our experiment, we used the saliency technique to highlight the marginal effect of a feature on the output with respect to the same input where that feature was removed to understand CNN inference.

The Occlusion Sensitivity method of Zeiler and Fergus [15] is based on dividing the input into segments called patches, masking them, and measuring the input impact of each defined patch on the classification results. For example, an image can be split into a grid of regular non-overlapping patches, and a mask can be slid over the image to cover the patches. The authors occluded different segments of an input image with a grey patch and visualised the change in the activations of the later layers. The output prediction performance drops significantly when the patch covers the critical area. A similar approach was proposed by Zhou et al. [16], where small grey squares were used to occlude image patches (in a dense grid) to explain scene classification. The visualisation shows the saliency regions of an image for its classification label. The Meaningful Perturbation method proposed by Fong and Vedaldi [17] uses the output value of the DNN, which changes when the input is penalised by deleting certain regions. Attribution aims to identify the regions of an image that are used to produce the output value. The idea is not to iterate over all possible perturbations but to search locally for the best perturbation mask, i.e., the smallest deletion mask. The authors considered three types of perturbations for creating a perturbation mask: replacing the input region with a constant value, injecting noise, or blurring the image. Extremal perturbations are regions of an input image that maximally affect the activation of a particular neuron in a DNN. The Extremal Perturbations method [18] optimises the perturbations by choosing smooth perturbations masks to maximise the confidence score of the classifier. Randomised Input Sampling for Explanation (RISE) [19] explains DNN black-box models by estimating pixel saliency importance (importance map) of input image regions. The importance of pixels is estimated by blurring them in random combinations, reducing their intensities to zero, and weighting their changes in the output by occlusion patterns. The authors [20] developed a fast pixel importance detection (saliency detection) method, the Universal Adversarial Perturbations method, for image classifiers by manipulating the results of the classifiers by masking salient parts of the input image. 

Of the above, the perturbation-based methods attempt to evaluate the importance of input segments, regions or pixels on the classifier’s decisions and determine how the deep network reacts to changes in the input. For example, if a certain part of the input is masked, how does it affect classification prediction. Thus, attribution evaluates the strength of the connection between the pixel or group of pixels and the specific network output. From this point, we can assume that certain regions of an image are not involved in the decision making of the classifier. This leads to an assumption that there might be parameters in the network that, when excluded from the deep network, keep the predictive precision of the classifier at the same level. 

In our experiment, we used the meaningful perturbation method to black out the region of a traffic sign in the feature map of the last fourth layer before a fully connected layer of a classification model of CNN PRYSTINE to distinguish the high- and low-impact influence parts of the CNN. Using the perturbation, we could have explained the influence of the fourth layer kernels on the classifier prediction. Thus, this approach allowed us to identify the vague kernels not involved in traffic sign and light classification and compressed them.

## 3. Implementation of Traffic Sign and Traffic Light Classifier

**Figure 1:**
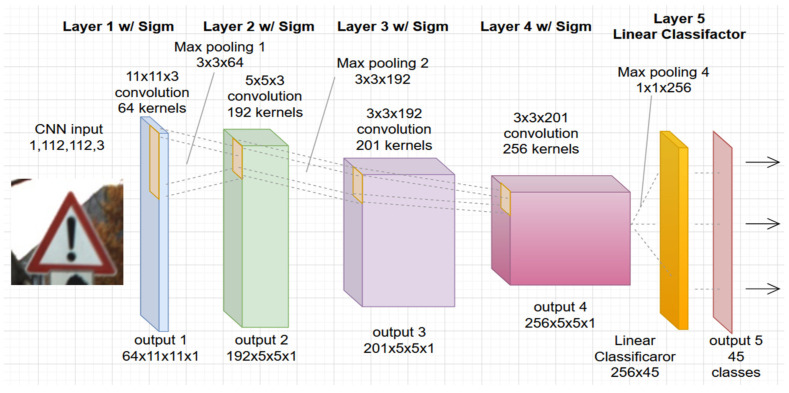
The architecture of the used 5-layer CNN road sign and light classification from the PRYSTINE project.

In this study, the authors used a convolutional network architecture developed specifically for the PRYSTINE project, see Figure 1. The project aims to realize Fail-operational Urban Surround network perception (FUSION) based on robust Radar and LiDAR sensors fusion and control functions to enable safe automated driving in urban and rural environments. The architecture of CNN PRYSTINE consists of five layers and is trained and tested with combined traffic sign and traffic light datasets [21].

Each layer of the network consists of convolution filtering, batch normalization, sigmoid activation, and downsampling by max pooling. The last, fifth layer, consists of a linear classifier with a softmax that produces the output of the network with 45 classes. The detailed definition and description with a code of CNN PRISTYNE can be found online at GitLab [22]. 

The network was trained and tested using a multi-class, single-image German Traffic Sign Recognition Benchmark (GTSRB) database [23]. The GTSRB contains more than 50,000 single images of traffic signs from 43 classes. The traffic signs were captured at different sizes and rotations, and with different light conditions, resulting in a natural distribution of road traffic signs. Next, the two classes of 2239 images from the LISA Traffic Light Dataset [24] were added to the GTSRB database (adding red/yellow light and green light classes). Finally, the CNN PRYSTINE model based on both the GTSRB and LISA datasets was trained.

Classification with CNN from the PRYSTINE project yielded a precision of 94.73% for traffic sign classification (tested only on the GTSRB test data) and 96.55% for traffic light classification (tested only on LISA test data). The execution time for one image in both classification categories was ~0.0187 s on Intel Xeon CPU. To test the explainability, a subset of 1000 images was randomly selected from the GTSRB test set and is available along with the developed code to test our experimental methodology.

## 4. CNN Layer Perturbation-Based Forward

**Figure 2:**
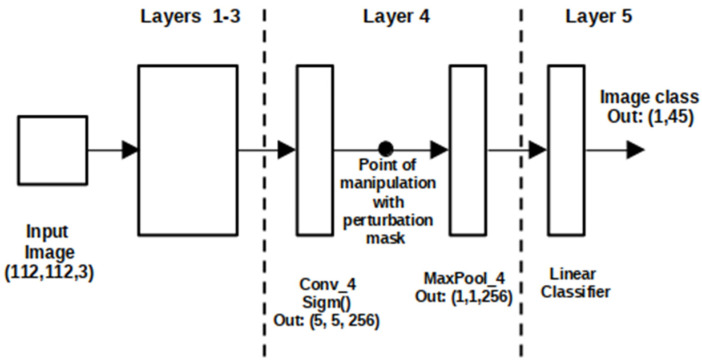
CNN PRYSTINE manipulations with perturbation mask in 4-layer architecture.

The standard perturbation approach aims at finding out which regions of an input image $x_{0}$ are used by the black box CNN f(x) to produce the output value $f\left(x_{0}\right)$. Derived from the meaningful perturbation method, given an input traffic sign image $x_{0}$ our goal was to define an explanation for traffic sign and traffic light classification in the output of the last convolution layer (fourth layer) before max-pooling by masking the output region *R* of the feature map with a constant black value; see Figure 2. 

Formally, let m : Λ→[0.1] be a perturbation mask, associating each pixel u∈ Λ with a scalar value m(u). Then the perturbation operator for a constant value is defined as [17]:

(1) $$\left[\Phi \left(x_{0};m\right)\right]\left(u\right)=m\left(u\right)x_{0}\left(u\right)+\left(1-m\left(u\right)\right)\mu_{0},$$

where $\mu_{0}$ is an average colour. We use $\mu_{0}=0$, which yields, after normalisation, the masking region R by substituting with black colour. Then output vector of the network’s fourth pooling layer $p_{4}$ are calculated as follows:

(2) $$p_{4}=pool_{4}(conv_{4}\left(p_{3}\right)),$$

where $conv_{4}()$ is an output of the convolutional fourth layer from the previous layers. The output vector of the network’s fourth pooling layer, including or excluding particular regions of an image from CNN decision making, is calculated as follows:

(3) $$p_{4}^{\prime }=pool_{4}(conv_{4}\left(p_{3}\right)*m)),$$

where m is a perturbation mask. Then, a coefficient $c_{r}\left(n\right)$ showing *n*-th feature involvement on CNN decision making of the *r*-th image (where *r* is an image number and in our experiment r=1000 of the test.py set), are calculated as follows:

(4) $$c_{r}\left(n\right)=\{\begin{array}{c} 1,\;if\;p_{4}\left(n\right)=p_{4}{\left(n\right)}^{\prime }\;\\ 0,\;\;\;\;\;\;\;\;\;\;\;\;\;\;\;\;\;\;if\;other \end{array}$$

Based on the feature involvement coefficient, the original CNN PRYSTINE model was compared with the model in which certain parts of the output of the final fourth convolutional layer of the network were excluded from the CNN traffic sign and traffic light prediction. In our experiment, the region of interest or mask R was a 3 × 3 region on a 5 × 5 feature map. This approach resulted in explaining the whole prediction power of the classification model. In our experiment, the perturbation mask filtered fourth layer kernels according to their significance. After the statistical significance of the feature was included in the decision making of the neural network, the kernels with the low impact of the fourth layer were removed from CNN PRYSTINE, resulting in the compression of the model. Thus, the model was compressed by pruning vague kernels accordingly to the precision calculations. The results of the experiments are presented in the next section. 

## 5. Experiments and Results

**Figure 3:**
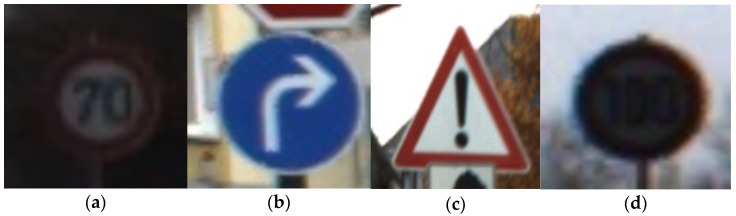
Examples of input images from the test dataset, where (**a**), (**b**), (**c**), traffic signs without occlusion, (**d**) occluded traffic sign.

This section provides details on the implementation of our experimental methodology. The code for all experiments, including a 500 image set for pruning and 500 images for testing the final result, is available online at GitLab. The code was developed using the *Python* programming language and the *TensorFlow 1.12* machine learning package. Examples of input traffic sign images from the used dataset are given in Figure 3.

We also included covered traffic signs to test the prediction quality of the CNN PRYSTINE classifier. The first three images in a row (Figure 3a–c) are real traffic signs and are correctly classified. In the false case, the (Figure 3d) image, the traffic sign was occluded (covered); as a result, the network did not predict it as a traffic sign. Thus, after the first step, based on how well our CNN PRYSTINE model classifies, we recognised that our network can provide correct predictions. Next, we wanted to check out explainability by using the meaningful perturbation method and find out how it behaves when the classifier kernels of the fourth layer of CNN PRYSTINE are pruned.

**Figure 4:**
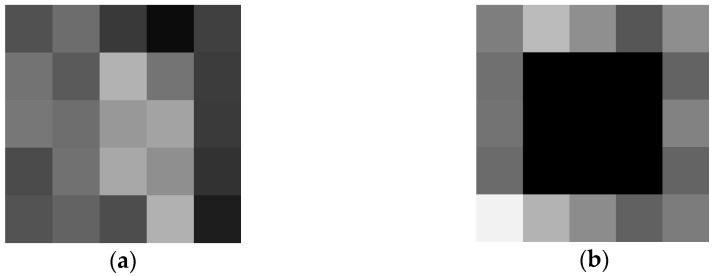
The output of the 4th layer feature map of CNN PRYSTINE before max pooling, (**a**) without masking, (**b**) with masking of the central region of 3 × 3 around the centroid.

The region of a 3 × 3 perturbation mask was applied onto the fourth layer feature map before max-pooling of a 1 × 1 × 1 × 256 array. Figure 4 depicts an example of the CNN PRYSTINE four-layer output of a 5 × 5 feature map, where Figure 4a is a feature map without a mask, but Figure 4b is a feature mask, where a mask replaces the centre of the 5 × 5 output with a constant pixel value of zeros (black colour). 

**Figure 5:**
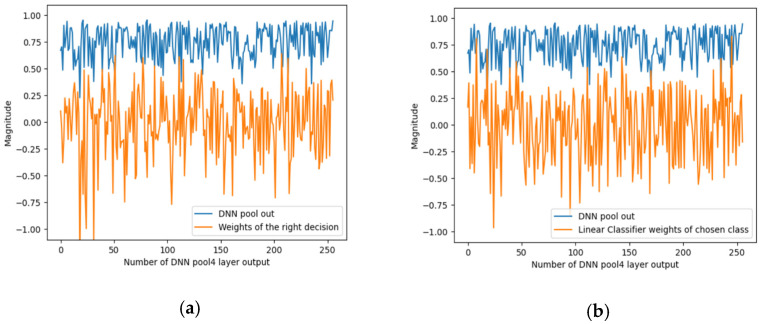
An example of the output signals of the DNN pool out from the 4th layer, representing the basic features in the DNN and linear classifier: (**a**) the corresponding signal/weights class that should have been correct, and (**b**) the most highly correlated signal/weights that were incorrectly selected as the correct class.

To summarise the effect of masking, to know the waveform of the signals to explain the predictions of the CNN PRYSTINE classifier (correct or incorrect), and to know the waveform of the signals that flow through the network model (the signals from the max-pooling layer of 1 × 1 of the output of the fourth layer) the signal characteristics were recorded; see Figure 5. It shows the characteristics of the signals for the case when the traffic sign is occluded. *DNN pool out* shows the values of the fourth layer sigmoid activation function for each of the 256 features. This means how much each feature is involved in an input image. 

Figure 5 below shows two options for the distribution of signals/weights. The first (Figure 5a) shows two selected classes of the linear classifier (LC) with softmax activation for the corresponding signal/weights of the correct decision. The second (Figure 5b) is the signal from the LC weights of the selected class. Both signals characterise the classification signals of the linear classifier that correlate most strongly with CNN PRYSTINE features. 

The experiment of explainability has shown that the low impact kernels are in fourth layer. When removing them, the processing and storage capacity improves the performance of a CNN PRYSTINE model. Results of the quality of compression are shown in Table 1, where the CNN compression rate $\alpha \left(M,M^{*}\right)$ of $M^{*}$ over M was calculated as follows:

(5) $$\alpha \left(M,M^{*}\right)=\frac{a}{a^{*}},$$

where a is the number of kernels in the original model (fourth layer, 256 kernels) and is that of the compressed model $a^{*}$. For instance, if eight vague kernels are pruned out of 256, the compression rate is 3.125%. 

As shown in Table 1, using the meaningful perturbation method, the performance of the CNN PRYSTINE network decreased by 1.4% when the compression rate was 3.125%, i.e., eight low-impact kernels were found and removed from the fourth layer. When the compression rate was 5.47% (14 kernels), the network performance slightly declined by 2.2% compared with the original CNN PRYSTINE classifier precision. When the compression rate of the fourth layer was 17.58% (45 kernels were pruned), the loss in precision rate was 6% compared to the original CNN PRYSTINE model. The proposed methodology is especially crucial when execution time and processing capacity are the main concerns. 

## 6. Discussion

Every model fails now and then, and when it does, we want to explain why it does. In the case of traffic sign and traffic light classification, where human lives are at stake, we face the problem of studying the inner workings of CNNs in addition to the classification. In this study we proposed a new methodology based on the XAI approach that “whitens” the black-box network. The results of our experimental methodology show that our traffic sign and traffic light image classification model, in which the vague kernels of the fourth layer were slightly compressed, can achieve the same precision as the state-of-the-art CNN models to some extent. Pruning 5% of network parameters results in a loss of only 2% in the precision of traffic sign and traffic light classification, which would be acceptable if one wants to achieve better performance in terms of processing capacity or execution time. However, in many cases, especially in Deep Learning, replacing an existing model with the pruned network elements leads to a trade-off in precision. This could be a self-defining goal if the model focuses on precision or needs to process faster. Another challenge is integrating explanations into an automated driving system to reduce its complexity and improve the model’s performance.

## 7. Conclusions

Many studies have been conducted to explain the decision-making process of CNNs for classification applications in computer vision. This work shows and experimentally proves that the CNN PRYSTINE model can be compressed using the meaningful perturbations mask. Using the proposed methodology, we achieved a 17% performance improvement with a precision loss of only 6%. However, compressing about 50% of the fourth convolution layer kernels would result in low precision of the network and thus lead to the low prediction power of traffic sign and traffic light classification. The classification precision could be tested and analyzed by using different perturbation methods on larger datasets and different DNN models. In addition, the classification precision could be adjusted by analysing the CNN kernels and comparing correct and error leading data subsets. Compression of the parameters of the different network layers will also prove useful. 

We aim to incorporate gradient-based explainability methods in our future works to identify and understand vague kernels for CNN prediction models. In addition, it is worth investigating whether kernel or parameter pruning is appropriate for network compression. Finally, we also aim to develop more sophisticated, robust and reliable explanatory algorithms to improve the prediction performance of classification models. Such algorithms could pave the way between the prediction structures of AI models and humans’ ground-truth knowledge.

## Figures and Tables

**Table 1:** Experimental results of compressing the CNN PRYSTINE 4th layer.

Compression Rate, %	Precision (Training Set of 500 Images on which Pruning is Made), %	Precision (Test Set of 500 Images), %
Original PRYSTINE CNN network	94.4%	94.4%
3.125%	94.4%	93.0%
5.47%	93.4%	92.2%
17.58%	89.2%	88.0%

## Data Availability

The used code is available at: http://git.edi.lv/kaspars.sudars/prystine_explainable_road_sign_classifier (accessed on 27 January 2022). The used GTRSB—the German Traffic Sign Benchmark database—is available at: https://www.kaggle.com/meowmeowmeowmeowmeow/gtsrb-german-traffic-sign (accessed on 27 January 2022). The used LISA Traffic Light Dataset data set is available at: https://www.kaggle.com/mbornoe/lisa-traffic-light-dataset (accessed on 27 January 2022).
